# Testing a videogame intervention to recalibrate physician heuristics in trauma triage: study protocol for a randomized controlled trial

**DOI:** 10.1186/s12873-016-0108-z

**Published:** 2016-11-11

**Authors:** Deepika Mohan, Matthew R. Rosengart, Baruch Fischhoff, Derek C. Angus, Coreen Farris, Donald M. Yealy, David J. Wallace, Amber E. Barnato

**Affiliations:** 1 Department of Critical Care Medicine, University of Pittsburgh, Room 638 Scaife Hall, 3550 Terrace Street, Pittsburgh, 15261 PA USA; 2 Department of Surgery, University of Pittsburgh, Pittsburgh, PA USA; 3 Department of Social and Decision Sciences, Carnegie Mellon University, Pittsburgh, PA USA; 4 RAND Corporation, Pittsburgh, PA USA; 5 Department of Emergency Medicine, University of Pittsburgh, Pittsburgh, PA USA; 6 Department of Medicine, University of Pittsburgh, Pittsburgh, PA USA

**Keywords:** Heuristics, Trauma triage guidelines, Physician decision making, Diagnostic error, Videogames

## Abstract

**Background:**

Between 30 and 40 % of patients with severe injuries receive treatment at non-trauma centers (under-triage), largely because of physician decision making. Existing interventions to improve triage by physicians ignore the role that intuition (heuristics) plays in these decisions. One such heuristic is to form an initial impression based on representativeness (how typical does a patient appear of one with severe injuries). We created a video game (*Night Shift)* to recalibrate physician’s representativeness heuristic in trauma triage.

**Methods:**

We developed *Night Shift* in collaboration with emergency medicine physicians, trauma surgeons, behavioral scientists, and game designers. Players take on the persona of Andy Jordan, an emergency medicine physician, who accepts a new job in a small town. Through a series of cases that go awry, they gain experience with the contextual cues that distinguish patients with minor and severe injuries (based on the theory of analogical encoding) and receive emotionally-laden feedback on their performance (based on the theory of narrative engagement). The planned study will compare the effect of *Night Shift* with that of an educational program on physician triage decisions and on physician heuristics. Psychological theory predicts that cognitive load increases reliance on heuristics, thereby increasing the under-triage rate when heuristics are poorly calibrated. We will randomize physicians (*n* = 366) either to play the game or to review an educational program, and will assess performance using a validated virtual simulation. The validated simulation includes both control and cognitive load conditions. We will compare rates of under-triage after exposure to the two interventions (primary outcome) and will compare the effect of cognitive load on physicians’ under-triage rates (secondary outcome). We hypothesize that: a) physicians exposed to *Night Shift* will have lower rates of under-triage compared to those exposed to the educational program, and b) cognitive load will not degrade triage performance among physicians exposed to *Night Shift* as much as it will among those exposed to the educational program.

**Discussion:**

Serious games offer a new approach to the problem of poorly-calibrated heuristics in trauma triage. The results of this trial will contribute to the understanding of physician quality improvement and the efficacy of video games as behavioral interventions.

**Trial registration:**

clinicaltrials.gov; NCT02857348; August 2, 2016.

## Background

Treatment at trauma centers improves outcomes for patients with moderate-to-severe injuries [1–3]. Professional organizations, state authorities, and the federal government endorse the systematic triage and transfer of these patients to trauma centers either directly from the field or after evaluation at a non-trauma center [4]. Nonetheless, between 30 and 40 % of patients with moderate-to-severe injuries still receive treatment at non-trauma centers (*under-triage)* [5–7]. The problem amplifies among the cohort evaluated by physicians at non-trauma centers—70 to 80 % of whom are under-triaged [8, 9].

Current efforts to change physician decision making in trauma triage focus primarily on education and outreach by opinion leaders [10]. These strategies assume that the problem is one of ignorance. In other words, physicians make the wrong decision because they either lack knowledge of the clinical practice guidelines or do not believe in the validity of the guidelines. However, this approach ignores the influence of intuitive judgments (*heuristics*) on triage decisions. Heuristics, mental short cuts based on pattern recognition, drive most decision making. They take precedence over rule-based algorithms (analytic cognitive processes), by springing to mind spontaneously and effortlessly. They work well under conditions of time-pressure and uncertainty precisely because they ignore irrelevant information and streamline decision making. Most importantly, they generate useful answers most of the time. However, they can also draw attention to the wrong contextual cues, leading to predictable errors of judgment (*biases*) [11–13]. Our observational and experimental work suggests that the use of heuristics may explain some patterns of non-compliance with clinical practice guidelines in trauma triage [14–16].

Because they are automatic, the use of heuristics cannot be eradicated. Moreover, the uncertainty and complexity of the clinical environment make well-calibrated heuristics a powerful tool [17]. We propose what we believe to be the first intervention designed to recalibrate physician heuristics. It draws on research from other domains suggesting the potential effectiveness of promoting *analogical encoding* and *narrative engagement*. Analogical encoding interventions use case studies to highlight core principles of decision making, emphasizing the identification of relevant and irrelevant contextual cues [18–21]. Narrative engagement interventions use messages designed to resonate with recipients in personally relevant and meaningful ways. Our intervention is an adventure video game that combines these two features [22, 23]. The planned study will compare the impact of the game on simulated physician trauma triage decisions with that of an educational program of equivalent duration.

## Methods

### Overview

We developed the video game (*Night Shift*) in collaboration with Schell Games (Pittsburgh, PA) through an iterative process involving behavioral scientists, cognitive psychologists, trauma surgeons, and emergency medicine physicians. We plan to compare the impact of *Night Shift* with that of a standard educational program on simulated triage decisions made by a convenience sample of emergency medicine physicians. We will recruit physicians working at non-trauma centers, randomize them to play *Night Shift* or to review a standard educational program, and assess their decision making on a validated virtual simulation.

The virtual simulation allows us to assess physician performance in terms of the number of severely injured patients not transferred to trauma centers (under-triage). We can also assess the effect of *Night Shift* on physician heuristics, by experimentally manipulating cognitive load in the simulation. Psychological theory predicts that cognitive load will increase reliance on heuristics, thereby increasing the under-triage rate in situations where those heuristics are inaccurate [24]. Our target heuristic is representativeness. In the context of trauma triage, it would entail forming an initial impression based on how typical a patient appears of the severely injured.

We hypothesize that: a) physicians exposed to *Night Shift* will have lower rates of under-triage compared to those exposed to the educational program, and b) cognitive load will not degrade triage performance among physicians exposed to *Night Shift* as much as it will among those exposed to the educational program.

### Participants

#### Study setting

Using a strategy that has proven successful in the past, we will recruit physicians at a national meeting of the American College of Emergency Physicians in the fall of 2016, using a booth in the Exhibition Hall [15, 16]. Physicians will be eligible for participation if they care for adult patients in the Emergency Department of either a non-trauma center or a Level III/IV trauma center in the United States. We will obtain verbal consent from eligible physicians, informing them that the study focuses on evaluating how best to disseminate clinical practice guidelines in trauma.

#### Randomization and blinding

**Fig. 1:**
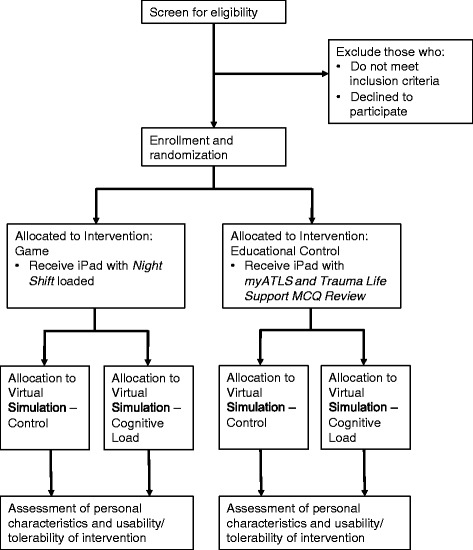
Overview of study protocol. Description of data: Flow diagram of recruitment and allocation strategy

We will randomize eligible physicians to complete (a) either *Night Shift* or the educational intervention, *myATLS and Trauma Life Support MCQ Review,* and (b) either the control or cognitive load version of a virtual simulation [Fig. 1]. We will generate a randomization scheme using Stata 13.0 (Statacorp, College Station, TX, USA) statistical software, using random block sizes of 4 or 8. After registering the participant, study personnel will obtain the intervention assignment from a central database. Although we cannot blind study personnel and participants to the intervention after allocation, we will mask group assignment during the analysis phase.

### Study protocol

Upon enrollment, subject physicians receive an iPad mini 2 with their intervention pre-loaded, along with written instructions on how to complete the study protocol. They will receive weekly reminder emails to complete the protocol for the duration of the study. We will ask participants to spend a minimum of one hour completing the intervention task. After completing it, they will be instructed to log in to a website that hosts: a) the virtual simulation; b) a questionnaire about their personal information, and c) a questionnaire about the intervention’s tolerability. Completing the simulation and two questionnaires will take approximately one hour. Participants will be able to complete the two portions of the study protocol at their convenience, within 1 month of enrollment. They will keep the iPad mini 2 as an honorarium (approximate value $260).

#### Intervention: night shift

The goal of Night Shift is to teach physicians to recognize patients with ‘non-representative’ severe injuries, defined as injuries that are classified by the American College of Surgeons as life-threatening or critical, but that do not fit the archetype for injuries requiring treatment at a trauma center [4]. The intervention is designed to increase analogical encoding by having players experience a case that goes awry because of reliance on the representativeness heuristic, and providing specific feedback on relevant and irrelevant cues (i.e. ones they might have missed and ones that might have misled them). It is designed to facilitate narrative engagement by the providing the information in an emotionally compelling way. After considering different types of games (e.g. puzzles, role-playing), we decided that an adventure video game would provide an environment realistic enough for existing triage guidelines to appear valid, while including an element of fantasy to promote character identification and engagement.

**Fig. 2:**
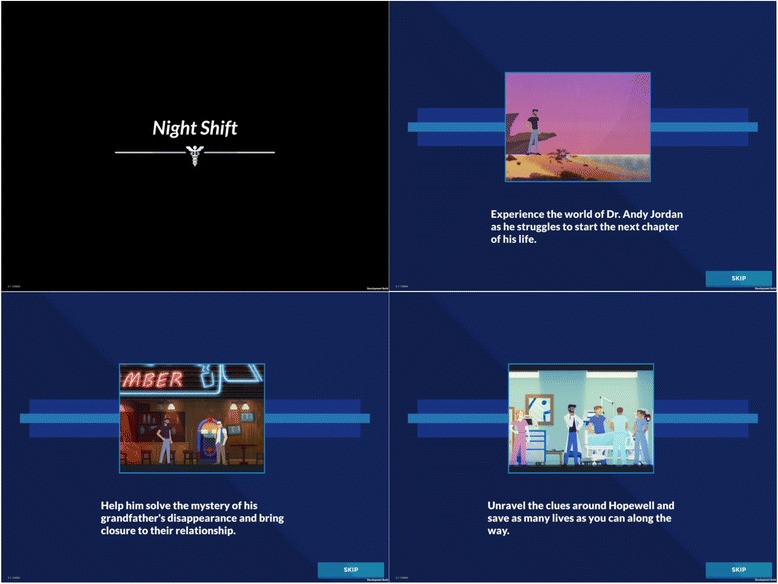
Screen shots of trailer to Night Shift. Description of data: We show the trailer to the game. We provided players with two explicit objectives in order to heighten narrative engagement, while simultaneously providing a vehicle for physician education

Players take on the persona of Andy Jordan, a young emergency medicine physician who moves home after the disappearance of his estranged grandfather (Robert Jordan) and takes a job in the local emergency department (ED). The introduction tells players that they have two objectives. The first is to diagnose and treat patients who present to their ED. The second is to solve the mystery of Robert’s disappearance: was he murdered or has he chosen to disappear? [Fig. 2].

The patients who present to the ED have both severe injuries and non-trauma complaints. In each case, subject physicians must interview the patients to elicit relevant elements of the history and physical exam. They then have the option of: a) ordering ancillary testing, b) discussing the case with a consultant, or c) discharging the patient home. Cases end when players make disposition decisions (admit, transfer, or discharge).

We categorize cases as ‘relevant’ or ‘non-relevant’ to our objective. ‘Relevant’ patients have non-representative severe injuries. When players fail to transfer one of these patients, he/she returns with complications that require additional treatment. Players also receive feedback on their performance from peers, family members, or their supervisor. The feedback includes both information about the clinical practice guidelines and a personal reprimand. In contrast, when players successfully transfer the patient to a trauma center, they receive an update, stating that the patient has improved, information about the guidelines, and a compliment on their diagnostic and decision-making capability.

‘Non-relevant’ patients either have representative severe injuries or non-trauma complaints. We included these cases because playtesters found that the ‘relevant’ cases did not pose sufficient diagnostic challenge, making game-play tedious. Players do not receive in-game feedback on their treatment of these patients. However, we do provide a summary of their performance at the end of *Night Shift*, which includes their ability to diagnose and treat the ‘non-relevant’ cases.

The mystery component of *Night Shift* occurs concurrently with the clinical challenges, and serves to facilitate players’ identification with their character and interest in their task. Players must solve Robert’s disappearance through interactions with other characters, including patients, and their physical environment. Andy Jordan’s background and character are also revealed through these interaction, which are designed to make him and his decisions more appealing and sympathetic [Table 1].

**Table 1:** Description of *Night Shift*

Duration: Three hours of gameplay possible.	
Objective: To change physicians’ archetype of a representative severe injury	
Conceptual framework	
	• Highlight relevant contextual cues within the context of a narrative to facilitate integration into a mental model of the decision problem (analogical encoding).
	• Provide personally-relevant and emotionally-compelling feedback that increases retention of the message (narrative engagement)
Premise: The player takes on the role of Andy Jordan, a young emergency medicine physician, who moves home after his grandfather’s disappearance and accepts a job at a local community hospital covering night shifts.	
Content	
	Medical: Physicians interview patients who present to the Emergency Department, and have the option of investigating further, discussing the case with a consultant, or discharging the patient home. The patients include: • 5 deceptive trauma cases with severe injuries adapted from clinical practice. These patients have injuries classifed by the American College of Surgeons as ‘life-threatening or critical’ but that might appear minor. They return with complications if under-triaged, and allow the player to gain experience with relevant contextual cues and receive to feedback on performance. • 5 non-trauma cases adapted from the clinical case records of the Massachusetts General Hospital, presented in the New England Journal of Medicine. These patients serve as a diagnostic challenge to facilitate player engagement in the clinical task. • 2 obvious trauma cases with severe injuries adapted from clinical practice. These patients serve as a management challenge to facilitate player engagement in the clinical task.
	Non-medical: Robert Jordan, Andy’s estranged grandfather, has disappeared. The prologue hints that his disappearance may or may not have occurred voluntarily. The player must solve the mystery by uncovering clues revealed through conversation with in-game characters and by exploring the environment.
Game mechanics	
	1. Connect the dots: clues (medical and non-medical) appear on a notepad on the screen. The player can draw connections between clues to uncover information and to unlock additional dialogue options.
	2. Tap to act: the player can tap on the screen to move through the world and interact with other characters. This mechanic also allows the player to perform key patient-care actions, including procedures like lumbar punctures and intubations.
	3. Points: players receive points for uncovering non-medical clues, which unlock in-game lore. Specifically, they can access letters written by Andy and his grandfather, which should provide additional insight into their characters and motivations.

#### Intervention: educational control

The gold standard educational strategy in trauma is *Advanced Trauma Life Support (ATLS)*. The American College of Surgeons have developed ATLS, a 2-day seminar, to teach participants: to assess the patient’s condition rapidly and accurately; to resuscitate and to stabilize the patient; to determine if the patient’s needs exceeds the capabilities of their facility; to arrange for the definitive care of the patient, and to ensure that optimal care if provided. The course includes lectures on key topics (e.g. assessing the severity of the injury), instruction on skills relevant to the initial stabilization of the patient (e.g. putting in chest tubes), and practice using standardized patients. Participants must complete a multiple choice test before and after the course to receive certification [25].

We will use a two-part educational program as a surrogate. First, physicians review the *myATLS* app, designed and produced by the American College of Surgeons as an adjunct to the ATLS course [26]. The app includes a review of each chapter of the ATLS textbook, a series of videos demonstrating common trauma procedures, and clinical resources including checklists for use at the bedside. Second, physicians use the *Trauma Life Support MCQ Review*, an app designed to help students prepare for the ATLS exam. It includes 550 multiple-choice questions with correct answers and explanations. We will ask physicians to review the *myATLS* app and then complete questions in the *Trauma Life Support MCQ Review*, spending at least 1 h on the combined tasks.

### Data sources and management

#### Interventions

We will install *Centrify* on each iPad that we distribute, an app that tracks usage of programs, to measure how long physicians spend on their assigned apps (adherence). Data from this app will download daily to a secure server hosted by the University of Pittsburgh. In addition, the *Night Shift* application collects data on each player’s behaviors and actions (e.g. disposition decisions) during game-play. These data will be summarized using Google Analytics and then downloaded to the server.

#### Outcome assessment

**Fig. 3:**
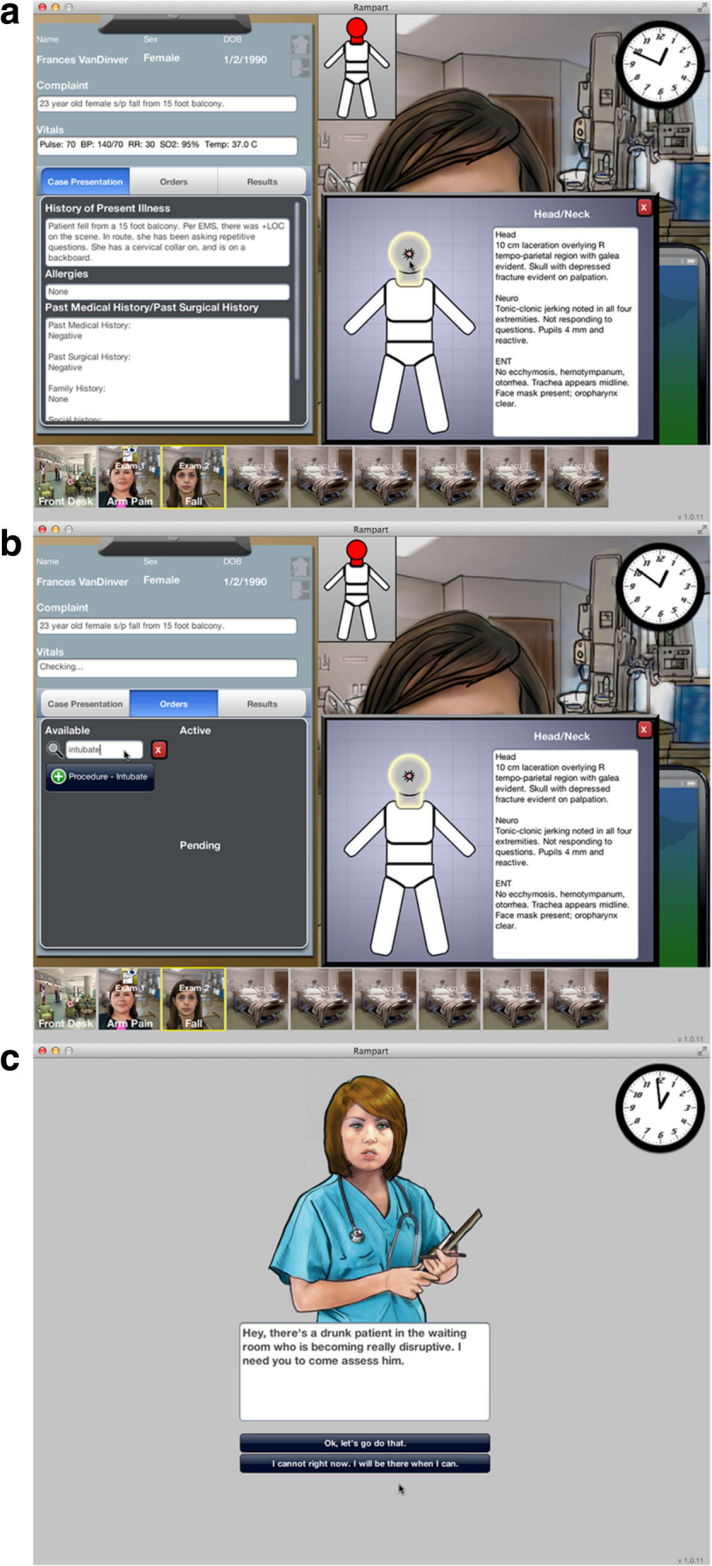
Screen shots of virtual simulation. Description of data: We show examples from the simulation. **a** Each case included a 2-D rendering of the patient, a chief complaint, vital signs, a history, and a written description of the physical exam. Physicians had 42 min (a simulated 8 h shift) to complete the ten cases. A clock at the top right of the screen helped track the passage of time. **b** Physicians could manage patients by selecting from a pre-specified list of 250 medications, studies, and procedures. **c** We included audio-visual distractors, including nursing requests for help with disruptive patients to increase the verisimilitude of the experience

After completing their assigned interventions, subjects will log into a website to complete a virtual simulation designed to replicate the task environment of an Emergency Department at a non-trauma center. The primary outcome of the clinical trial will be performance on the simulation, measured as the physician’s rate of under-triage (proportion of severely injured patients not transferred to a trauma center). We previously collaborated with a gaming company (Breakaway Ltd; Hunt Valley, MD) to develop a 2-D simulation to assess physician decision making in trauma triage [Fig. 3]. The simulation has both internal reliability as well as construct validity [16]. Most importantly, in prior studies, we found that physicians make similar decisions for trauma patients on the simulation as they do in real-life. Performance on the virtual simulation will be transmitted from the website to a secure server hosted by the University of Pittsburgh.

The simulation has ten cases: three severely injured patients (one representative, two-non-representative), three minimally injured patients, and four non-trauma cases. Users must evaluate and manage these cases over 42 min, simulating a busy 8-h ED shift. New patients arrive at pre-specified (but unpredictable) intervals, so that physicians must manage multiple patients concurrently. Each case includes a 2-D rendering of the patient, a chief complaint, vital signs which update every 30 s, a history, and a written description of the physical exam. In the absence of clinical intervention by the player, severely injured patients and critically ill distractor patients decompensate and die over the course of the simulation.

Physicians manage patients by selecting from a pre-specified list of 250 medications, studies, and procedures. Some orders affect patients’ clinical status, leading to corresponding changes in their vital signs and physical exam. Other orders generate additional information, presented as reports added to the patients’ charts. The cases end when physicians either make a disposition decision (admit, discharge, transfer) or the patient dies.

There are two versions of the simulation: a control and a cognitive load condition. As mentioned earlier, cognitive load is expected to increase reliance on heuristics [24]. When poorly calibrated, these heuristics result in predictable errors. By varying cognitive load, we can assess the effect of the interventions on physicians’ heuristics (a secondary outcome measure). Specifically, a comparison of the difference in performance under control and cognitive load conditions of physicians in the two arms of the study will provide insight into the mechanism by which the interventions work. If physicians exposed to *Night Shift* demonstrate both lower under-triage rates, and less effect of cognitive load on performance, we can infer that *Night Shift* functions by recalibrating heuristics. In contrast, if physicians exposed to *Night Shift* perform better in general but have the same rate of errors when working under cognitive load as physicians exposed to the educational module, then we can infer that *Night Shift* functions through some alternative mechanism.

We operationalize cognitive load in two ways. First, we vary the complexity of the non-trauma cases. In the control arm, non-trauma cases have routine complaints (e.g. appendicitis), arrive hemodynamically stable, and do not deteriorate over the course of the simulation. In the cognitive load arm, non-trauma cases are critically ill (e.g. have sepsis), arrive hemodynamically unstable, and deteriorate without adequate management. Second, we reduce the number of rooms that physicians can use to evaluate patients, from eight in the control arm to four in the cognitive load arm, which increases the number of distractors that they receive.

#### Questionnaire to assess personal characteristics

Each physician will complete a questionnaire querying age, sex, race, ethnicity, educational background (board certification, ATLS certification, years since completion of residency). We will also assess individual differences that might vary between the two samples, and might influence the efficacy of the different interventions. Physicians will complete the Big Five Inventory-10, a ten-item instrument that measures personality traits [27].

#### Questionnaire to assess usability and tolerability of interventions

To measure the tolerability of the interventions, we will ask physicians to provide qualitative feedback about the experience. Additionally, we will ask physicians who use Night Shift to complete the Narrative Engagement Scale developed and validated by Busselle and Bilandzic [28]. The instrument has 12 items that measure narrative understanding, attentional focus, narrative presence, and emotional engagement. It will allow us to explore causes for *Night Shift*’s success or failure at changing physician decision making, in future analyses.

### Analyses

We will calculate the response rate as the proportion of enrolled physicians who start to use their assigned intervention, and the completion rate as the proportion who finish the virtual simulation. We will include physicians in the analysis who do not spend one hour on their assigned intervention (i.e. evaluating it in terms of intention-to-treat). We will exclude physicians who do not complete the virtual simulation (i.e., those for whom we have missing outcome data).

We will summarize physician characteristics (including personality traits) using means (standard deviations) for continuous variables and proportions (%) for categorical variables, and will compare the distribution of characteristics between the two intervention groups using Students *t*-test and chi-squares as appropriate.

#### Physician performance

We hypothesize that: a) physicians exposed to *Night Shift* will have lower rates of under-triage compared to those exposed to the educational program, and b) cognitive load will not degrade triage performance among physicians exposed to *Night Shift* as much as it will among those exposed to the educational program.

To test these hypotheses, we will first score participants’ performance on each trauma case based on a review of their disposition decisions, using American College of Surgeons guidelines as the gold standard [4]. We will calculate an under-triage rate for each physician:

$$ \frac{\mathrm{number}\ \mathrm{o}\mathrm{f}\ \mathrm{severely}\ \mathrm{injured}\ \mathrm{patients}\ \mathrm{not}\ \mathrm{t}\mathrm{ransferred}\ \mathrm{t}\mathrm{o}\ \mathrm{t}\mathrm{rauma}\ \mathrm{centers}}{\mathrm{total}\ \mathrm{number}\ \mathrm{o}\mathrm{f}\ \mathrm{severely}\ \mathrm{injured}\ \mathrm{patients}} $$

We will assess the influence of the intervention (game or educational program), cognitive load (absent or present), and the interaction between these factors on physicians’ under-triage rates using an ANOVA.

#### Adherence

We will summarize the length of time that physicians in each group spend on their intervention and compare the duration using Students t-tests. Information about adherence will provide insight into the tolerability of the two interventions and will also allow for exploratory analyses into the mechanism of the interventions’ success or failure.

#### Tolerability

We will categorize qualitative feedback as positive or negative and will compare the tolerability of the two interventions. Among physicians who play *Night Shift*, we will also summarize responses to the Narrative Engagement Scale.

### Human subjects and power calculation

We have designed the experiment to capture a moderate effect (one-half a standard deviation) of the interventions on the difference between the under-triage rates of physicians who complete the game and educational program with an alpha of 0.05 and a power of 80 % [29]. Based on prior studies, we anticipate a 70 % response rate and will recruit 366 physicians [15, 16].

### Security, ethics, and dissemination

#### Data security

On enrollment in the trial, participants will receive a unique identifier. They will use that identifier to login to *Night Shift* (if assigned to that condition) and to the website that hosts the virtual simulation and questionnaires. Only the primary investigator (PI) will have access to the linkage file connecting the identifier to the physician’s name and contact information. This file will be encrypted and stored on a secure server at the University of Pittsburgh.

#### Dissemination of results

Results from the study will be reported to the public through manuscripts and oral presentations at national meetings. We will provide an abstract of the findings to all participants. Access to the de-identified dataset will be made available upon written request to the study team.

## Discussion

The objective of this research program is to develop a video game intervention that will improve physicians’ triage decisions by engaging them in cases where generally useful heuristic decision rules lead to errors in judgment. *Night Shift* provides explanations of appropriate decisions, presented in an intuitive way, so as to facilitate integration with their normal thought processes. We will use a randomized controlled trial to compare the efficacy of that video game with that of a standard educational control intervention of similar duration and complexity.

The National Academy of Medicine recently issued a white paper, *Improving Diagnosis in Health Care*, in which it highlighted the influence of heuristics on diagnostic error [30]. Existing interventions have had limited success at ensuring that physicians use well-calibrated heuristics [31]. These efforts typically focus on increasing physicians’ use of rational processes either implicitly by disseminating rule-based algorithms or explicitly by promoting reflective reasoning. The latter encourages physicians to take a step back, to consider their diagnoses more carefully, and to recognize the shortcomings of judgments made based on intuition [32–34]. A few studies recommend removing the clinician altogether from the decision problem, shifting the burden of judgment to decision tools or external consultants [35, 36]. All of these proposals ignore the potentially adaptive nature of heuristics. Although heuristics can result in predictable errors, they arise because they often produce useful responses to complex questions with relatively little cognitive effort. An intervention that can improve physician heuristics would leverage the strengths and talents of the people at the center of the diagnostic challenge: physicians [37].

Our intervention focuses on the representativeness heuristic, whereby individuals’ initial judgment of a case reflects how typical it seems of a process. In the trauma triage setting, reliance on representativeness would lead to correct diagnoses of, say, cases involving penetrating injuries, but not cases involving seemingly more benign processes (e.g. falls for elderly individuals). The intervention uses stories designed to immerse participants in playing the role of a physician concurrently solving both clinical and personal problems. It draws on three bodies of research finding the potential power of stories to facilitate behavioral change. One research thread finds that stories facilitate processing and retaining new data, called narrative engagement [22]. In our context, the stories are meant to help physicians integrate their simulated experience into the mental models evoked in normal clinical practice. The second body of research finds that practicing desired behaviors in a safe environment helps people to gain warranted feelings of self-efficacy, providing the confidence needed to deploy newly acquired skills [22]. The third body of research finds that stories can engage players cognitively and emotionally in ways that transcends traditional forms of education [38, 39].

We also exploit the growing appeal and prevalence of serious game technology. Video games are a $22 billion/year industry. 155 million Americans play video games. The average gamer is 34 years old. Twenty-seven percent of players are older than 50; 44 % are female. A majority (80 %) of US households own a device to play games [40]. Statistics do not exist on the number of physicians who play video games. However, states and professional organizations already require between 20 and 50 h a year of continuing medical education as a condition for licensure. Games could easily become part of the roster of accepted educational activities.

This study has potential limitations including its design as a two-arm trial. An alternative design would include four arms: control, game, educational module, game plus educational module. However the constraints of the budget and concerns about recruiting sufficient numbers of physicians precluded this approach. Second, we plan to use a convenience sample to test the efficacy of the game, which may not represent the population at-large. If the game does affect performance, we can then proceed with testing its effectiveness. However, if we find no effect even among motivated participants, it provides a rationale to explore other methods of promoting behavior change. Third, we have chosen an educational intervention without any proven effectiveness. However, the structure (a didactic program that informs users of the clinical practice guidelines accompanied with multiple choice questions to reinforce key decision priniciples) reflects that of ATLS, the current gold standard for continuing medical education in trauma. Furthermore, the American College of Surgeons promotes *myATLS* as an adjunct to ATLS, giving the intervention additional face validity.

In conclusion, physician heuristics play an important role in diagnostic error. Altering heuristics is a challenge, with no effective solution. Serious games have attributes that make them well suited to address this problem, and therefore offer a new approach. We have chosen the special case of trauma triage to test the effect of a video game intervention to recalibrate heuristics. The results of this trial will contribute to the literature on physician quality improvement and the efficacy of video games as behavioral interventions.

Trial Status: Recruiting

## Abbreviations

ANOVA: Analysis of variance
ATLS: Advanced Trauma Life Support
ED: Emergency Department
